# On the relationship between foveal mask interference and mental imagery in peripheral object recognition

**DOI:** 10.1098/rspb.2023.2867

**Published:** 2024-03-13

**Authors:** Giulio Contemori, Carolina Maria Oletto, Luca Battaglini, Marco Bertamini

**Affiliations:** Department of General Psychology, University of Padova, Padova, Italy

**Keywords:** vision, perception, mental imagery, psychophysics, foveal feedback, visual cortex

## Abstract

A delayed foveal mask affects perception of peripheral stimuli. The effect is determined by the timing of the mask and by the similarity with the peripheral stimulus. A congruent mask enhances performance, while an incongruent one impairs it. It is hypothesized that foveal masks disrupt a feedback mechanism reaching the foveal cortex. This mechanism could be part of a broader circuit associated with mental imagery, but this hypothesis has not as yet been tested. We investigated the link between mental imagery and foveal feedback. We tested the relationship between performance fluctuations caused by the foveal mask—measured in terms of discriminability (d′) and criterion (C)—and the scores from two questionnaires designed to assess mental imagery vividness (VVIQ) and another exploring object imagery, spatial imagery and verbal cognitive styles (OSIVQ). Contrary to our hypotheses, no significant correlations were found between VVIQ and the mask's impact on d′ and C. Neither the object nor spatial subscales of OSIVQ correlated with the mask's impact. In conclusion, our findings do not substantiate the existence of a link between foveal feedback and mental imagery. Further investigation is needed to determine whether mask interference might occur with more implicit measures of imagery.

## 1. Introduction

According to the foveal feedback hypothesis, the foveal cortex may serve as an auxiliary computational unit in peripheral visual discrimination [1]. Presentation of a foveal mask with a slight delay can influence peripheral discrimination performance, likely through interference with foveal feedback. The impact of the foveal mask depends on its congruence with the target, enhancing performance for congruent masks and impairing it for incongruent ones [2]. The (timing of) maximal foveal mask interference can be delayed if an additional imagery task, such as mental rotation, is required before object discrimination [3].

The foveal cortex is theorized to play a role in preserving and manipulating task-relevant information [4,5]. Introduction of foveal noise can alter the mental representation of peripheral stimuli, impacting both discriminability and decision criterion [6]. One possible explanation could be temporal proximity, causing information pooling between the mask and peripheral targets, hindering the mental representation of the stimuli [6,7]. While the mask interference has been consistently observed at the group level, individual-level variability is large. We suggest that this variability may be attributed to individual differences, such as the vividness of mental imagery. This study investigates the relationship between mental imagery and foveal feedback by correlating variations in discriminability (d′) and decision criterion (C), induced by foveal masks, with questionnaire scores. To date, the connection between foveal feedback and explicit mental imagery has not been explored [8].

### (a) The foveal feedback

Recent findings suggest that the foveal retinotopic cortex is involved in processing peripheral information (for a review, see [9]). For instance, a study using transcranial magnetic stimulation (TMS) found that disrupting foveal processing after, but not during, stimulus presentation, negatively affects performance [10]. Similar results were obtained using a foveal mask and variable stimulus onset asynchronies (SOAs), showing a performance decline when masks were presented 100–300 ms after stimulus onset (for a review, see [8]).

One explanation is that the foveal cortex acts as an auxiliary computational unit for processing fine details in the peripheral visual field, relying on feedback signals from higher-level areas [1]. Another explanation is that this feedback serves as a predictive mechanism to aid future foveation and is related to saccade planning [11,12]. However, the delay in foveal feedback due to concurrent mental operations cannot be explained solely by the foveation theory [3]. Functional magnetic resonance imaging (fMRI) studies have shown a correlation between information in the foveal retinotopic cortex and task performance in peripheral discrimination, emphasizing the role of feedback without subsequent foveation [1].

Regarding the nature of information relayed to the foveal cortex, the foveal mask does not affect performance in tasks involving discrimination of low-level features or blurred stimuli [2,3]. Notably, the impact of the foveal mask varies depending on its properties, with congruent masks enhancing performance and incongruent masks reducing it [2,12]. This effect was not observed with Gabor patches. These findings suggest that the feedback mechanism is more active during challenging tasks requiring detailed shape processing [2]. Furthermore, the foveal mask affects colour discrimination when both the target and mask are coloured but not when the mask is greyscale. In shape-related tasks, both coloured and greyscale masks yield similar disruptive effects [13]. Additionally, the foveal mask hampers subordinate category discrimination more than supraordinate categories [14].

It is hypothesized that, in some conditions, the brain has access only to summary statistics about shape. However, the extraction of summary statistics presents both advantages and disadvantages [15]. Factors like spatial [15] or temporal proximity [16] can interfere with this processing, affecting recognition. In the context of foveal feedback, even when targets and masks are spatially distanced, temporal proximity could lead to features pooling. In a peripheral same/different task with a delayed foveal mask, this pooling could induce a perceptual bias towards perceiving peripheral targets as different. This concept aligns with a conservative bias in the decision criterion [6]. According to signal detection theory, the bias represents the individual decision threshold used to categorize stimuli as signal or noise. Participants were found to adopt a more conservative criterion (more ‘different' responses) when the foveal mask was presented approximately 174 ms after target display, with a sensitivity dip around 94 ms [6]. This suggests that the foveal mask may interfere at different stages, with the earlier stage related to perceptual representation and the later stage linked to decision-making.

The timing of the mask's effect may vary based on the mental operations required before comparing the targets, with more extensive mental rotation causing a greater delay in mask effectiveness [3]. This is not due to a general increase in task difficulty but rather to a combined effect of the mental operations needed to complete the task [7]. However, other factors influence the timing of the mask's impact. Notably, prior research has reported a wide-ranging timing of the peak mask effect, varying from 50 to 350 ms [8]. This variability can be attributed partly to differences in experimental designs but also to individual differences. To illustrate this point, we reanalysed data from Contemori *et al*. [6] with the aim of examining the frequency of individual minimum d′. The results of this reanalysis are shown in the electronic supplementary material. The reanalysis reveals that the timing of the most pronounced disruptive effect of the foveal mask (electronic supplementary material figure S1) varies not only between different experiments but also among individual participants. In our previous study, some individuals were significantly affected by the mask, while others showed no substantial decline in performance.

Until now, this variability had not been systematically investigated and had been addressed through an extended pre-test practice phase [3].

In summary, information conveyed to the foveal cortex plays a crucial role in decision-making and the mental manipulation of previously seen objects [3]. The theory suggests that foveal feedback involves the high-resolution foveal cortex processing extra-foveal shape details [8], likely enhancing perceptual decision precision [4,5]. Considering significant individual differences in factors such as vividness, precision, strategy and expertise, we hypothesize that part of the variability in the foveal mask effect may be linked to individual differences in representing stimuli in the mental space [17].

### (b) Mental imagery

Mental imagery involves consciously generating mental images, either intentionally, as in daydreaming or problem-solving, or automatically, as when reading a descriptive passage [18]. The extent of automaticity varies among individuals, with some having more vivid and involuntary mental imagery. Aphantasia, on the other hand, impairs voluntary image creation but imagery may still manifest during dreams [19].

Visual imagery can be conceptualized either as a unified, single ability or as a multidimensional construct. Kosslyn [20] proposed breaking down visual imagery into subprocesses, including generation, maintenance and transformation processes [20,21]. Tasks that implicitly assess visual imagery ability, such as those based on mental rotation, hold a distinctive position bridging subjective and objective measures of imagery. Mental rotation entails encoding the representation of an object (generation) and rotating it (transformation) to align with another presented view [22]. The mental rotation tasks in Fan *et al*. [3], like the original design by Shepard & Metzler [22], do not require verbal reports or explicit instructions for imagery utilization. These tasks occupy an intermediate space between conscious imagery and the sketchpad proposed in working memory models, as indicated by prior research linking mental rotation skills with working memory capacity [23–25]. Despite theoretical distinctions, visual imagery and visual working memory share similarities, making a clear separation challenging [26].

Notably, differences in mental imagery ability extend beyond variations in vividness. In 2005, Kozhevnikov, Kosslyn and Shephard introduced the object–spatial–verbal cognitive style model, which identifies three dimensions: object imagery (used by visualizers who primarily rely on mentally depicting objects), spatial imagery (used by visualizers who primarily rely on mentally manipulating and navigating objects in space), and verbal cognitive style (used by verbalizers who primarily rely on verbal–analytical strategies).

Earlier research emphasized the proficiency of ‘object visualizers' in tasks involving vivid visualization of pictorial properties, while ‘spatial visualizers' excelled in spatial imagery tasks such as mental rotation [27–29]. These cognitive styles vary among individuals and are linked to their daily activities. For instance, scientists and engineers tend to excel in spatial imagery, while visual artists lean towards object imagery [28].

Proficient imagers, compared to those with moderate or limited imagery skills, may use imagery as a memory aid for visual working memory tasks [30]. Salge *et al*. [31] found that individuals with vivid mental imagery are more susceptible to perceiving illusory faces in noise, known as pareidolia. Dijkstra *et al*. [32] discovered that engaging in mental imagery leads to a more liberal criterion for detecting external stimuli, even when the imagined and actual stimuli do not match. Internally generated sensory signals during imagery can sometimes be confused with perceptual input. In a related study using a contrast detection task, congruent imagery shifted the point of subjective equality (PSE) leftward without impacting the just noticeable difference (JND) compared to non-congruent imagery [33].

In these examples, imagination precedes the stimulus appearance. In foveal feedback, the stimulus precedes the generation of the non-retinotopic mental image, later disrupted by the mask. We hypothesize that, in both scenarios, the foveal visual cortex, influenced by both feedback and feedforward information, temporally integrates imagined and visual stimuli.

### (c) The foveal sketchpad

The foveal retinotopic cortex may serve as a tool for preserving and manipulating task-relevant information, akin to Baddeley's visuospatial sketchpad (VSSP) as discussed by Oletto *et al*. [8]. Visualizers may be particularly reliant on this mechanism compared to verbalizers. The introduction of foveal noise may disrupt the mental image of the stimuli, altering the decision criterion [6,7], but it can also enhance performance when the mask is congruent to peripheral stimuli [2,12].

Perception and mental imagery share neural activity along the ventral pathway [34]. These common representations exhibit a neural signature within the alpha frequency band, originating from parieto-occipital sources [35]. Wilming *et al*. [36] discovered a dissociation between gamma and alpha frequency bands in perceptual decision-making. Gamma conveyed stimulus-dependent information, while alpha carried endogenous information related to perceptual choice. Studies on mice show that the primary visual cortex is involved in both feedforward and feedback processes at distinct time intervals [37].

These findings suggest a temporal alternation between feedback and feedforward processes, possibly involving overlapping neural populations but at different times [38]. Foveal neurons may cyclically contribute, following individual alpha rhythms, to feedback in the mental representation of visual stimuli and to a perceptual decision-making process [6]. When reimagining stimuli for discrimination tasks, it is advantageous to discard positional information causing a potential competition for foveal neural resources.

### (d) Aim and design

The investigation aimed to explore the association between mental imagery and foveal feedback. Fan *et al*. [3] demonstrated that the introduction of a mental rotation task resulted in a delay in the effect of the mask, indicating a connection between foveal feedback and mental imagery. Our aim was to examine the correlation between variations in performance induced by the foveal mask—as assessed by decrements in d′ scores and shifts in C—and scores derived from two questionnaires designed to assess mental imagery. Two contrasting scenarios were anticipated:

(a) Susceptibility: individuals with superior visual imagery might experience increased susceptibility to the disruption caused by the foveal mask, leading to a negative correlation between imagery scores and performance reduction.
(b) Resilience: individuals with superior visual imagery might demonstrate heightened resilience to the disruptive effects of the foveal mask, resulting in a positive correlation between imagery scores and mask-related declines in d′.

Prior research has shown that the foveal mask biases decision-making, likely due to the temporal pooling of information between the mask and targets [6,7]. Although Fan *et al*. [3] lacked criterion data, we hypothesized similar scenarios for C as for d′. A null result would ultimately challenge the notion of a direct link between foveal feedback and imagery.

In addition, we investigated the correlation between sensitivity (d′) and mental imagery, independently of mask timing. The hypothesis suggested that individuals with more vivid imagery would exhibit better overall performance. Alternatively, d′ and C might correlate with the individual's cognitive style.

Vividness, which encompasses liveliness, clarity, sharpness, and distinctiveness of mental images [39], can be evaluated using the Vividness of Visual Imagery Questionnaire (VVIQ), a self-report questionnaire developed by Marks in 1973. The VVIQ assesses an individual's capacity to generate and experience vibrant mental images. Participants are presented with different scenarios and are instructed to rate the clarity and vividness of the images they can conjure in each scenario [40].

The Object–Spatial Imagery and Verbal Questionnaire (OSIVQ), another self-report instrument, assesses imagery across three specific dimensions postulated by Blazhenkova & Kozhevnikov in 2009. In previous studies, the VVIQ demonstrated good psychometric properties [41] but showed no correlation with other implicit measures of imagery [42]. Conversely, OSIVQ scores notably correlated with performances on various implicit tasks including mental rotation [27–29].

The combined use of these two questionnaires covers a broad spectrum of imagery subcomponents, investigating different aspects of visual imagery. Moreover, these questionnaires are quick to administer without unduly extending the overall experiment duration.

Previous research suggests that among the three subscales of the OSIVQ, the object-oriented subscale is most strongly correlated with the VVIQ [27]. This subscale is also closely linked to the visual aspect of mental imagery, which we expect to be most influenced by the foveal mask. Therefore, we anticipated that the mask's effect would be specific to the object-oriented subscale and would align with the VVIQ. However, existing literature has identified correlations between spatial-oriented imagery and mental rotation [27–29]. Given that the study of Fan *et al*. [3] was based on mental rotation, a correlation between the spatial subscale of OSIVQ and d′ and C should also be considered. We did not expect a correlation between the verbal subscale and the dip in d′ caused by the mask. Additionally, we extended these hypotheses to the C parameter.

To investigate the association between the d′ dip/C peak and questionnaire scores, we calculated individual variation in d′ values for each participant at two time points: 100 ms and the time corresponding to their specific performance minimum. The inclusion of the 100 ms SOA was driven by previous research findings where the group average minimum d′ occurred around 94 ms [6].

However, considering the variability in timing, relying solely on the 100 ms for correlation with questionnaires might be too rigid. To account for this, we conducted two additional correlations. The first one examined the correlation between the d′ values at individually determined performance minima and questionnaire scores. The second correlation assessed the relationship between the corresponding timing of these minima and questionnaire scores. Similar correlation analyses were performed for the C parameter. Contemori *et al*. [6] found a peak at 174 ms. Therefore, we selected a 150 ms SOA for correlation with the questionnaire scores, along with the individual C maxima and their timing.

The hypotheses and methodology of this study were preregistered and can be found on Open Science Framework (OSF): https://osf.io/tkmwv. Data and analysis are available on OSF: https://osf.io/rjdgb/.

## 2. Methods

The study employed an experimental design derived from previous research [3,6,7]. Participants engaged in a ‘same/different' task with peripheral stimuli followed by a central dynamic mask. The stimulus-onset asynchronies (SOAs) ranged from 0 to 400 ms in 50 ms intervals, alongside a baseline no-noise condition. The study involved 40 conditions in a 2 × 2 × 10 factorial design (target type, stimulus position, and SOA). Each SOA was repeated 60 times. Participants, recruited between September 2022 and July 2023, included 64 participants (40 females), aged 19–39, with normal or corrected-to-normal vision. Ethical approval (protocol number 4812) was obtained from the Psychology Ethics Committee at the University of Padua. Data collection consisted of 38 400 trials, with no performance threshold set. The experiment adhered to the Declaration of Helsinki.

Participants watched a video introducing the task and stimuli, followed by the same/different judgment task. The experiment took about 90 min and included 600 trials divided into three blocks, preceded by an 80-trial practice block. During the practice block, visual feedback for correct responses was provided.

The experiment was generated using PsychoPy3 [43]. Stimuli were displayed on an Eizo ColorEdge CS2420 with gamma correction, 1920 × 1200-pixel resolution, 60 Hz, and 61.1 cm diagonal size. Each participant sat in a dimly lit room, approximately 57 cm from the screen, using a chin rest. An eye tracker (Gazepoint GP3) ensured fixation within 2° of the central point. Each trial presented two targets for 100 ms, followed by a dynamic 7 × 7° coloured mask for 83 ms at various SOAs, including a baseline no-noise condition. Participants responded after a 600 ms delay.

**Figure 1.**
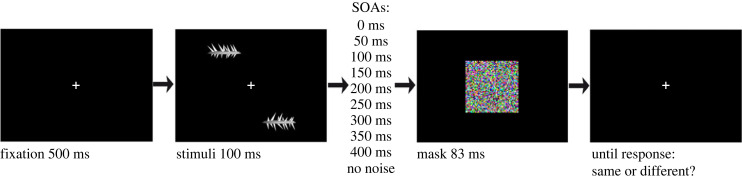
Schematic of a trial in the experiment. Two spiky shapes appeared briefly for 100 ms in opposite corners of the visual field, differing in length and orientation of upper and lower spikes. A dynamic noise mask appeared in the central vision for 83 ms, with one of nine possible time intervals: 0, 50, 100, 150, 200, 250, 300, 350 and 400 ms. The baseline condition had no mask. Each trial randomly selected two shapes from a pool of 1296 possible shapes, with an equal chance of them being ‘same' or ‘different'.

Stimuli were abstract three-dimensional shapes provided by courtesy of the authors, varying in spike length and orientation [3]. The stimuli had an average size of 3 × 1.5° of visual angle and were presented at an eccentricity of 7° (figure 1). After the task, participants completed two questionnaires, with no time limit. They were encouraged to close their eyes and immerse in the described situations while responding. The questionnaires were administered in Italian [44] for all but two participants, who completed the English versions. Specifically, 62 participants received the VVIQ in Italian, while 57 participants were administered the OSIVQ in Italian. The English versions were given to two participants for each questionnaire.

Similar to previous studies [3,6,7], participants with d′ < 0.7 were excluded from group analysis related to the mask effect. Conversely, for correlational analysis, we did not enforce a minimum performance threshold. Individuals with lower overall performance provide useful extremes, indicative of aphantasic or hyperphantasic traits. Consequently, separate criteria were applied for group and correlational analyses.

Data analysis was performed in R [45] and included calculating sensitivity (d′) and criterion (C) using signal detection theory. Fixed effect in linear mixed models was assessed via a type III F-test using the Satterthwaite approximation for degrees of freedom. We conducted orthogonal contrasts between SOAs to validate the presence of performance dips/peaks. Post hoc comparisons were used to pinpoint the minimum/maximum. These contrasts were set up to compare the SOA marginal means from the model against the baseline no-noise condition. For statistical robustness, 95% confidence intervals were adjusted using Bonferroni and *p*-values for the *z*-test were corrected with the false discovery rate (FDR).

To examine the associations between individual average d′ and C across SOAs alongside questionnaire scores, Spearman's rank-based coefficients were employed. Additionally, we explored correlations involving the variation in d′ at 100 ms and the individual minimum, the variation in C at 150 ms and the individual maximum, and questionnaire scores. To separate the impact of overall performance from mask-related effects, we calculated the difference between baseline d′ and d′ at 100 ms, as well as the participant's absolute minimum. A similar subtraction was conducted for C. Using no noise as baseline isolated the impact induced by the mask from participant performance level. This approach does not alter the timing of the minimum. For all correlations, significance was set at 0.05. Owing to the numerous correlations (24 in total), the probability of at least one significant *p*-value by chance alone—if all null hypotheses were true—was 0.7. Therefore, we should expect about one false positive [46]. More detailed information about data analysis and methods is in the electronic supplementary material.

## 3. Results

### (a) Mask effect on d′

The mean d′ value across all conditions was 1.31 (95% CI [1.27, 1.35]). After excluding participants with d′ < 0.7, the mean d′ value increased to 1.38 (95% CI [1.33, 1.42]). Analyses in this section were conducted—as in previous studies—on the sample with d′ > 0.7, *n* = 58.

The linear mixed model analysis revealed a substantial overall explanatory power (conditional *R*^2^ = 0.49), with marginal effects (marginal *R*^2^) accounting for 0.04 of the variance. The intercept of the model, corresponding to SOA = 0, was estimated at 1.36 (95% CI [1.26, 1.45], *t*_511_ = 28.42, *p* < 0.001). Within the model, the linear effect of SOA was statistically significant and positive (*β* = 0.28, 95% CI [0.18, 0.37], *t*_511_ = 5.79, *p* < 0.001; std. *β* = 0.55, 95% CI [0.36, 0.73]). However, the quadratic, cubic, and higher-degree effects of SOA were non-significant. Type III F-test, with the levels of the SOAs as the only within-subjects variable, revealed a significant main effect of medium size (*F*_8, 456_ = 4.49, *p* < 0.001; *η*^2^(partial) = 0.07, 95% CI [0.03, 1.00]). Post hoc comparisons indicated that four SOA levels differed significantly from the no-noise baseline condition in the range between 0 and 250 ms with the minimum d′ being at 50 with an estimate of 1.23 (*t*_184_ = −5.066, *p* < 0.001). The results of the comparisons are summarized in table 1.

**Table 1.** Contrasts for the d′ at each SOA level against the baseline no mask condition. Results are given on the response scale (d′). The null hypothesis (null) is the average d′ in the no mask baseline condition. Confidence level used: 0.95. Conf-level adjustment: Bonferroni method for nine estimates. *P*-value adjustment: FDR method for nine tests. Lowest d′ SOA = 50 ms, mean = 1.229.

SOA (ms)	mean d′	s.e.	d.f.	lower.CL	upper.CL	null	*t*-ratio	*p*-value
0	1.25	0.0654	184	1.07	1.43	1.56	−4.755	<0.001***
50	1.23	0.0654	184	1.05	1.41	1.56	−5.066	<0.001***
100	1.26	0.0654	184	1.08	1.44	1.56	−4.589	<0.001***
150	1.31	0.0654	184	1.12	1.49	1.56	−3.858	<0.001***
200	1.38	0.0654	184	1.20	1.56	1.56	−2.751	0.0117*
250	1.38	0.0654	184	1.20	1.57	1.56	−2.691	0.0117*
300	1.43	0.0654	184	1.25	1.61	1.56	−2.013	0.0512
350	1.43	0.0654	184	1.24	1.61	1.56	−2.047	0.0512
400	1.53	0.0654	184	1.35	1.71	1.56	−0.455	0.6494

**Figure 2.**
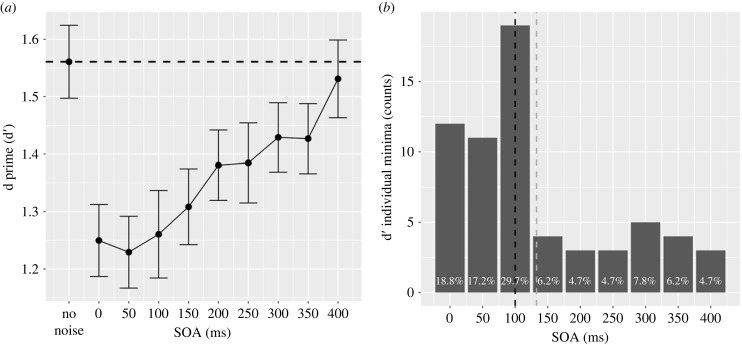
The figure shows d′ as a function of SOA (no noise, 0, 50, 100, 150, 200, 250, 300, 350, 400 ms). In (*a*), black dots represent mean values with standard errors. The black dashed line represents the baseline no-noise condition. In (*b*), bars represent the frequency plot of d′ individual minima. Black and grey dashed lines represents the median and the mean timing, respectively.

Data indicate a drop in performance for SOAs lasting between 0 and 250 ms with the lowest point at 50 ms. d′ data are shown in figure 2.

**Figure 3.**
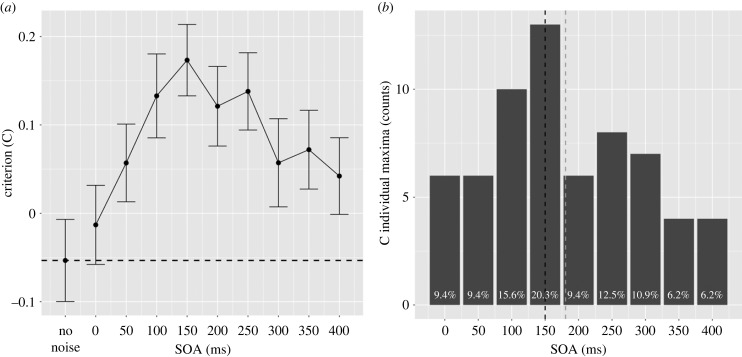
The figure shows C as a function of SOA (no noise, 0, 50, 100, 150, 200, 250, 300, 350, 400 ms). In (*a*), black dots represent mean values with standard errors. The black dashed line represents the baseline no-noise condition. In (*b*), bars represent the frequency of C individual maxima. Black and grey dashed lines represents the median and the mean timing, respectively.

The median of the individual minima d′ values was 0.739, with a corresponding 95% confidence interval (CI) of [0.632, 0.895]. Similarly, the mean of the individual minima d′ values was 0.753, with a 95% CI of [0.655, 0.860]. The distribution of the timings for the individual minima is presented in figure 3. Notably, the median timing for the individual minima equalled 100 ms, 95% CI [100, 100]. The mean timing was estimated to be 132.813 ms, with a 95% CI of [100.781, 159.375].

### (b) Mask effect on criterion

For the criterion, the mean value across all conditions was 0.11 (95% CI [0.08, 0.14]). After excluding participants with d′ < 0.7, the mean decreased to 0.07 (95% CI [0.04, 0.10]). Analyses in this section were also conducted—as in previous studies—on the sample with d′ > 0.7, *n* = 58.

The model total explanatory power was substantial (conditional *R*^2^ = 0.67), with marginal effects (marginal *R*^2^) accounting for 0.03 of the variance. The intercept of the model, corresponding to SOA = 0, was estimated at 0.09 (95% CI [0.01, 0.16], *t*_511_ = 2.30, *p* = 0.022). Within the model, the linear effect of SOA was statistically significant and positive (*β* = 0.01, 95% CI [−0.04, 0.06], *t*_511_ = 0.39, *p* = 0.693; s.d. *β* = 0.03, 95% CI [−0.12, 0.18]), quadratic trend was statistically significant and positive (*β* = −0.14, 95% CI [−0.19, −0.09], *t*_511_ = −5.51, *p* < 0.001; s.d. *β* = −0.42, 95% CI [−0.56, −0.27]), cubic trend was statistically significant and positive (*β* = 0.06, 95% CI [0.01, 0.11], *t*_511_ = 2.42, *p* = 0.016; s.d. *β* = 0.18, 95% CI [0.03, 0.33]). The fourth degree polynomial trend, and higher-degree effects of SOA were non-significant. Type III F-test, with the levels of the SOAs as the only within-subjects variable, revealed a statistically significant and medium effect (*F*_8,456_ = 5.09, *p* < .001; *η*^2^(partial) = 0.08, 95% CI [0.03, 1.00]). Pairwise comparisons indicated that all SOA levels differed significantly from the no-noise baseline condition except SOA 0. The peak in C was at 150 ms SOA with an estimate of 0.173 (*t*-ratio = 5.051, *p* < 0.001). The results of the pairwise comparisons are summarized in table 2.

**Table 2.** Contrasts for the criterion at each SOA level against the baseline no-mask condition. Results are given on the response scale (C). The null hypothesis (null) is the average C in the no-mask baseline condition. Confidence level used: 0.95. Conf-level adjustment: Bonferroni method for nine estimates. *P*-value adjustment: FDR method for nine tests. Highest C SOA = 150 ms, mean = 0.173.

SOA (ms)	mean C	s.e.	d.f.	asymp.LCL	asymp.UCL	null	*t*-ratio	*p*-value
0	−0.013	0.045	113	−0.140	0.114	−0.053	0.897	0.372
50	0.057	0.045	113	−0.070	0.184	−0.053	2.459	0.020*
100	0.133	0.045	113	0.006	0.260	−0.053	4.150	<0.001***
150	0.173	0.045	113	0.046	0.300	−0.053	5.051	<0.001***
200	0.121	0.045	113	−0.006	0.248	−0.053	3.889	<0.001***
250	0.138	0.045	113	0.011	0.265	−0.053	4.263	<0.001***
300	0.057	0.045	113	−0.070	0.184	−0.053	2.462	0.020*
350	0.072	0.045	113	−0.055	0.199	−0.053	2.793	0.011*
400	0.042	0.045	113	−0.085	0.169	−0.053	2.128	0.040*

Data indicate a positive shift in C for SOAs lasting between 50 and 400 ms with the high point at 150 ms. C data are shown in figure 3.

The median of the individual maxima C values was 0.422, with a corresponding confidence interval (CI) of [0.384, 0.489]. Similarly, the mean of the individual maxima C values was 0.420, with a 95% CI of [0.344, 0.499]. The distribution of the timings for individual maxima is presented in figure 3. Notably, the median timing for the individual maxima equalled 150 ms, and the 95% CI for this measurement ranged from [100, 150]. The mean timing was estimated to be 180.469 ms, with a 95% CI spanning from [150, 208.583].

### (c) Correlation between mental imagery questionnaires and average d′ and C

The correlations between mental imagery questionnaires and the average d′ and C values are reported in this section. In contrast to previous analyses, we included participants with d0 < 0.7. Sixty-four completed the VVIQ and 59 who also completed the OSIVQ. As expected, Spearman's rank correlation (*ρ*) between VVIQ and the OSIVQ object subscale was positive, statistically significant and large (*ρ* = 0.37, *S* = 21 670.13, *p* = 0.004), confirming previous findings [27,47]. No significant correlations were found between VVIQ and the verbal and spatial subscales. Similarly, the correlations between the three subscales of OSIVQ did not yield significant results.

**Figure 4.**
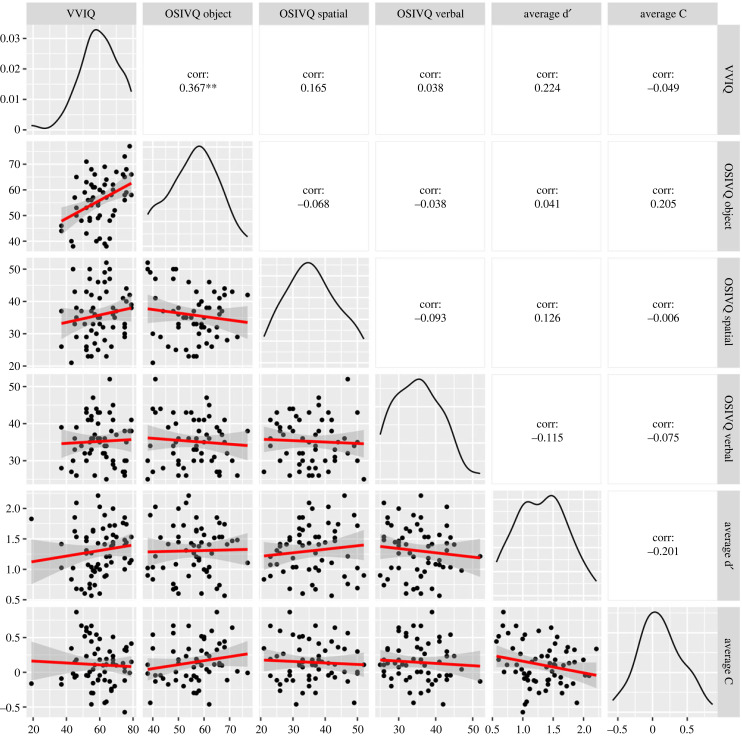
Scatter plot matrix. The matrix shows how all the possible pairs of variables were related to each other. On the top of the diagonal, the value of the Spearman correlation (*ρ*). On the diagonal, the distribution of each variable. On the bottom of the diagonal, the bivariate scatter plots with linear fits are displayed.

The correlation between d′ and the scores of all questionnaires did not yield significant results. Additionally, the correlation between C and the scores of all questionnaires did not produce significant results. A visual inspection revealed the presence of some possible extreme data points particularly in the VVIQ, as depicted in figure 4. As a precautionary measure, we conducted a reanalysis of the correlations, excluding individuals beyond 2.5 s.d. from the mean for each variable. This led to the removal of two data points, one for the VVIQ with a value of 19 and one for the OSIVQ verbal with a value of 52. The overall pattern of correlations remained largely consistent, except for the correlation between VVIQ and average d′, which, in this instance, exhibited a positive, statistically significant association of medium strength (*ρ* = 0.27, *S* = 30 307.00, *p* = 0.031). The figure following the removal of outliers is provided in the electronic supplementary material.

### (d) Correlation between mental imagery scores and discriminability (d′) variation at 100 ms and at individual minima

We initially calculated the magnitude of variation in d′ by subtracting the no-noise d′, from the d′value at 100 ms and at the participant's absolute minimum in the range 0–400 ms. The timing of the individual minimum in d′ corresponds to the SOA at which the participant's d′ reached the absolute minimum.

**Figure 5.**
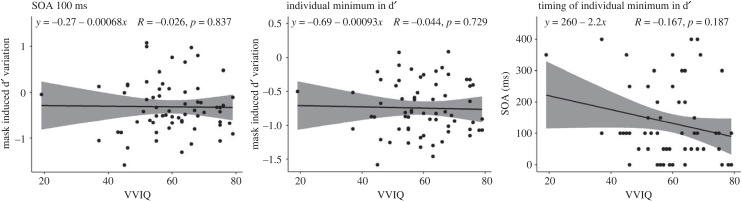
Correlation between VVIQ and d′. The left panel displays the correlations at 100 ms, the central panel shows correlations at individual minima and the right panel represents correlations with the timing of the individual minima. The bands indicate 95% confidence intervals.

The correlation between VVIQ and d′ variation at 100 ms and at individual minima did not yield significant results. Specifically, Spearman's rank correlation (*ρ*) between VVIQ and d′ variation at 100 was negative, statistically not significant and tiny (*ρ* = −0.03, S = 44 822.15, *p* = 0.837). Similarly, the correlation between VVIQ and d′ variation at the individual minima was negative, statistically not significant and tiny (*ρ* = −0.04, *S* = 45 610.95, *p* = 0.729). Moreover, the correlation between VVIQ and timing of the individual minima was negative, statistically not significant, and small (*ρ* = −0.17, *S* = 50 974.90, *p* = 0.187). These three correlations are presented in figure 5.

The correlation between OSIVQ object and d′ variation at 100 ms and at individual minima did not yield significant results. Specifically, the correlation between OSIVQ object and d′ variation at 100 ms was negative, statistically not significant, and tiny (*ρ* = −0.04, *S* = 35 456.98, *p* = 0.786). The correlation between OSIVQ object subscale and d′ variation at individual minima was negative, statistically not significant, and small (*ρ* = −0.18, *S* = 40 359.85, *p* = 0.174), and the correlation between OSIVQ object and timing of individual minima was negative, statistically not significant, and very small (*ρ* = −0.07, *S* = 36 613.87, *p* = 0.599).

The correlation between OSIVQ verbal and d′ variation at 100 ms was negative, statistically not significant and small (*ρ* = −0.14, *S* = 39 007.59, *p* = 0.291), the correlation between OSIVQ verbal and d′ variation at individual minima was negative, statistically significant, and medium (*ρ* = −0.28, *S* = 43 679.96, *p* = 0.034), and the correlation between OSIVQ verbal and timing at individual minima was negative, statistically significant, and large (*ρ* = −0.32, *S* = 45 002.18, *p* = 0.015).

**Figure 6.**
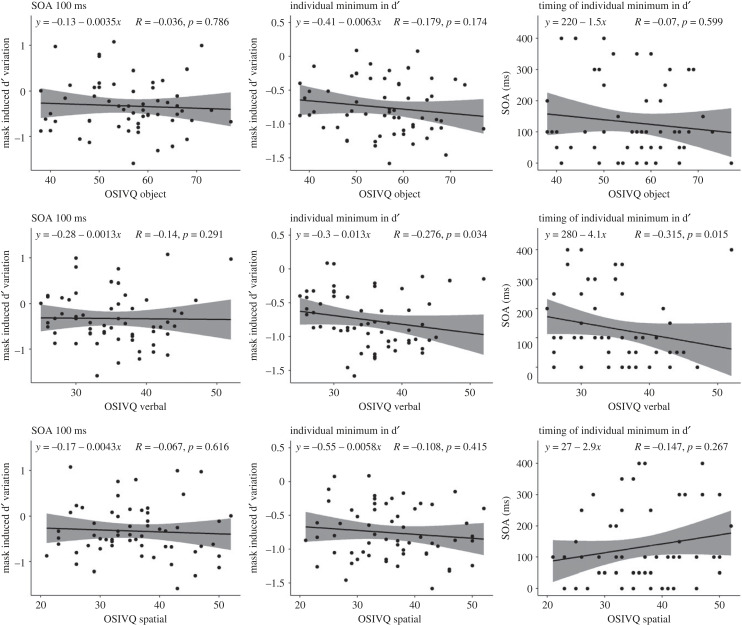
Correlation between OSIVQ and d′. The left column displays the correlations at 100 ms, the central column shows correlations at individual minima and the right column represents correlations with the timing of the individual minima. The upper row displays the correlations with the object subscale, the central row shows correlations with the verbal subscale and the lower row represents correlations with the spatial subscale. The bands indicate 95% confidence intervals.

The correlation between OSIVQ spatial and d′ variation at 100 ms was negative, statistically not significant and very small (*ρ* = −0.07, *S* = 36 502.90, *p* = 0.616), the correlation between OSIVQ spatial and d′ variation at individual minima was positive but statistically not significant and small (*ρ* = 0.14, *S* = 29 500.10, *p* = 0.298) was negative, statistically not significant and small (*ρ* = −0.11, *S* = 37 919.71, *p* = 0.415), and the correlation between OSIVQ spatial and timing at individual minima was positive, statistically not significant, and small (*ρ* = 0.15, *S* = 29 190.50, *p* = 0.267). These nine correlations are presented in figure 6.

### (e) Correlation between mental imagery scores and criterion (C) variation at 150 ms and at individual maxima

**Figure 7.**
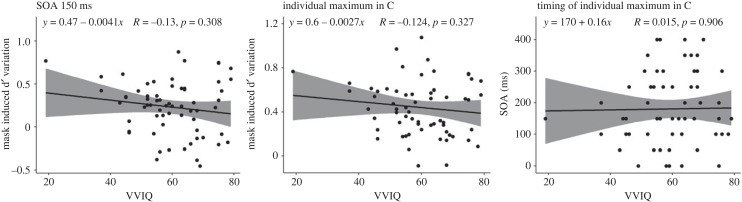
Correlation between VVIQ and C. The left panel displays the correlations at 150 ms, the central panel shows correlations at individual minima and the right panel represents correlations with the timing of the individual minima. The bands indicate 95% confidence intervals.

We calculated the magnitude of variation in C by subtracting the participant's no-noise C, from the C value at 150 ms and at the participant's absolute maximum in the range 0–400 ms. The timing of the individual maximum in C corresponds to the SOA at which the participant's C reached the absolute maximum. The correlation between VVIQ and C variation at 150 ms and at individual minima did not yield significant results. Specifically, Spearman's rank correlation (*ρ*) between VVIQ and C variation at 150 ms was negative, statistically not significant and small (*ρ* = −0.13, *S* = 49 337.70, *p* = 0.308). Similarly, the correlation between VVIQ and C variation at the individual maxima was negative, statistically not significant and small (*ρ* = −0.12, *S* = 49 116.48, *p* = 0.327). Moreover, the correlation between VVIQ and the timing of the individual maxima was positive, statistically not significant and tiny (*ρ* = 0.02, *S* = 43 020.32, *p* = 0.906). These three correlations are presented in figure 7.

The correlation between OSIVQ object, spatial and verbal subscales, and C variation at 150 ms and at individual minima is not significant. The correlation between OSIVQ object subscale and C variation at 150 ms was negative, statistically not significant and tiny (*ρ* = −4.09 × 10^−3^, *S* = 34 360.11, *p* = 0.975). The correlation between OSIVQ object subscale and C variation at individual maxima was negative, statistically not significant and very small (*ρ* = −0.06, *S* = 36 238.59, *p* = 0.657). The correlation between OSIVQ object subscale and timing at individual maxima was positive, statistically not significant and small (*ρ* = 0.18, *S* = 27 942.26, *p* = 0.164).

The correlation between OSIVQ verbal and C variation at 150 ms was positive, statistically not significant and very small (*ρ* = 0.08, *S* = 31 617.79, *p* = 0.567), the correlation between OSIVQ verbal and C variation at individual maxima was negative, statistically not significant and small (*ρ* = −0.16, *S* = 39 756.09, *p* = 0.221), and the correlation between OSIVQ verbal and timing at individual maxima was negative, statistically not significant and very small (*ρ* = −0.07, *S* = 36 469.94, *p* = 0.621).

**Figure 8.**
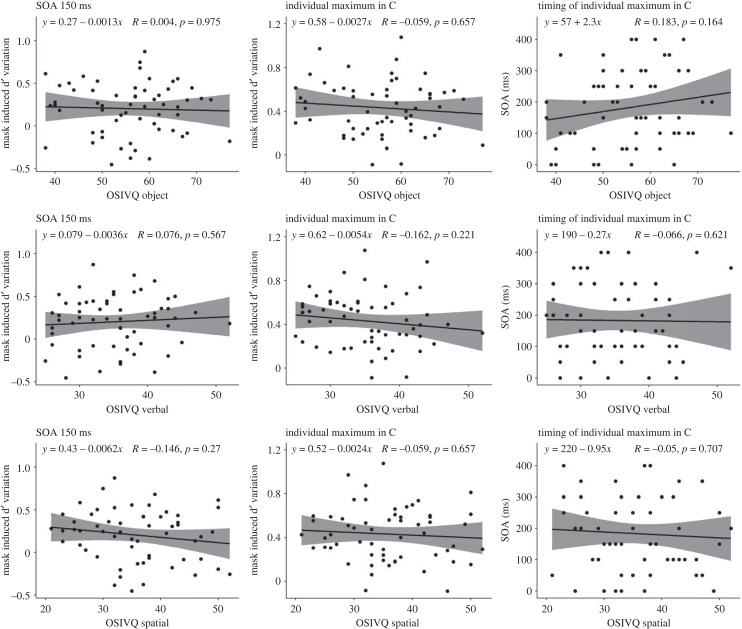
Correlation between OSIVQ and C. The left column displays the correlations at 150 ms, the central column shows correlations at individual minima and the right column represents correlations with the timing of the individual minima. The upper row displays the correlations with the object subscale, the central row shows correlations with the verbal subscale and the lower row represents correlations with the spatial subscale. The bands indicate 95% confidence intervals.

The correlation between OSIVQ spatial and C variation at 150 ms was negative, statistically not significant and small (*ρ* = −0.15, *S* = 39 219.36, *p* = 0.270), the correlation between OSIVQ spatial and C variation at individual maxima was negative, statistically not significant and very small (*ρ* = −0.06, *S* = 36 239.57, *p* = 0.657), and the correlation between OSIVQ spatial and timing at individual minima was negative, statistically not significant and very small (*ρ* = −0.05, *S* = 35 932.57, *p* = 0.707). These nine correlations are presented in figure 8.

## 4. Discussion

This study explored the connection between mental imagery and foveal feedback in a visual discrimination task. To do this, we analysed participants' scores on two mental imagery questionnaires in relation to their ability to discriminate peripheral stimuli. Peripheral discrimination tasks were conducted with and without a foveal mask, presented within a 0–400 ms timeframe. We calculated average d′ and C values for each participant and correlated them with scores from the VVIQ and OSIVQ subscales. Additionally, we examined individual d′ and C values at specific time points: 100 ms for d′, 150 ms for C, and the time point corresponding to the lowest d′ or highest C values, correlating these with questionnaire scores.

Our findings support the foveal feedback hypothesis, showing that the mask impacted discriminability and response criteria. Notably, when the mask appeared between 0 and 250 ms, accuracy decreased, with the most significant effect at 50 ms after target. However, frequency analysis showed that most participants exhibited their minimum d′ at 100 ms. These results are consistent with previous studies indicating that visual masking interferes with processing peripheral stimuli during this critical time windows [3,6].

Our analysis revealed that participants became more conservative in their responses, resulting in more ‘different' responses, when the mask appeared 150 ms after the target stimuli. This aligns with Contemori *et al*. [6] who found a C peak at around 174 ms. This implies that the mask not only affects perceptual discriminability but also exerts influence over participants' decision-making, possibly due to increased uncertainty.

Unlike previous findings [6], our study indicates that the linear model provides a better explanation of the effect of SOA than a quadratic or higher-degree model. This disparity may be attributed to the lower number of trials per participant, necessary for the inclusion of mental imagery questionnaires, which could have elevated intraindividual variability. This highlights the fragility of the foveal mask's effect and its susceptibility to individual differences.

Initially hypothesizing that individual differences in mental imagery might account for this variability, we considered two scenarios: individuals with higher imagery vividness being less affected by the mask (resilience hypothesis) or individuals with higher imagery vividness experiencing increased interference from the mask due to reliance on this ability (susceptibility hypothesis). We considered the former scenario more likely.

Given that peripheral discrimination requires high-detail (pictorial) processing, we expected a negative correlation between the variation in d′ associated with the mask and the OSIVQ object score. This hypothesis was substantiated by the observation that foveal feedback is more active during challenging tasks requiring detailed shape processing and object identification [2,3,14]. Conversely, considering that the original task in Fan *et al*. [3] entailed mental rotation, and mental rotation has been shown to correlate with OSIVQ spatial [27–29], a correlation with this subscale would also be plausible.

Contrary to expectations, our data did not support either prediction for d′. Hypotheses related to C were also not confirmed. Despite a more pronounced shift in criterion associated with the mask compared to its impact on discriminability, no support was found for its association with mental imagery.

After eliminating an outlier with a VVIQ score of 19, which likely represented the sole aphantasic individual in our sample, we observed a discernible relationship between the VVIQ score and the individual average d′. It is worth noting that aphantasia is commonly characterized by a VVIQ score below 32 [19], although consensus on this threshold is lacking. Despite this tenuous link between the vividness of mental imagery and peripheral discrimination ability, we found no correlation between mask-related variation in discriminability or C and VVIQ, OSIVQ object or OSIVQ spatial.

We did find two significant correlations for OSIVQ verbal: one with the individual minimum, and one with the timing of the individual minimum in d′, although these correlations are hard to interpret. We had no predictions about correlations between OSIVQ verbal and d′. It could be a false positive due to multiple tests. On the other hand, these correlations may suggest a difference between verbalizers and visualizers. Verbalizers, owing to their limited reliance on visual mental imagery, might encounter reduced interference from the mask. This could lead to a lower and earlier decline in performance, likely attributed to attention interference rather than feedback interference, given the early timing.

These findings alone are inconclusive when considered alongside the overall pattern of null results from other correlations. Consequently, the origin of high individual variability remains unclear and requires further investigation. It remains plausible that a correlation exists between the measured effects (d′ drop/C high) and aspects of visual imagery not captured by VVIQ or OSIVQ.

Our study drew inspiration from the work of Fan *et al*. [3], which employed mental rotation, a spatial reasoning task. While this task implicitly assesses visual imagery, there are instances in the literature indicating preserved mental rotation ability in individuals with aphantasia [48,49]. Moreover, spatial reasoning is strongly linked to working memory [23–25]. Our study deliberately diverged from Fan *et al*. [3] to explore how individual differences in vividness and imagery profiles affect foveal feedback. While the questionnaires primarily focused on conscious and voluntary mental imagery, it is conceivable that the mask's intrusion interferes with more automatic processes, such as iconic or working memory. Our study did not directly assess working memory, which previous research has suggested is related to visual imagery and perceptual discrimination [50]. Therefore, the hypothesis of a connection between peripheral stimuli, the foveal mask, and working memory remains a topic for future research.

A limitation of our study is that, although we largely succeeded in replicating findings using the foveal masking paradigm, the best-fitting model for the effect of SOA on d′ was linear, not quadratic as expected based on previous results [6,7]. It is possible that increasing the number of practice and/or testing trials could yield a clearer measure of the mask's effect and consequently larger correlations with imagery indices.

## 5. Conclusion

Our study confirms that a foveal mask affects perceptual discriminability and response bias in a peripheral discrimination task. We tested the role of visual imagery and found that, at the level of individual differences, there was no clear link between visual imagery and the effect of the foveal mask. This study represents the initial exploration of the link between non-retinotopic foveal feedback and imagery. Future research should incorporate measures of working memory and implicit imagery into the experimental paradigm.

## Data Availability

The hypotheses and methodology of this study were preregistered and can be found on Open Science Framework (OSF): https://osf.io/tkmwv. Data and analysis are available on OSF: https://osf.io/rjdgb/. Supplementary material is available online [51].
